# Platelet Polyphosphate Signals Through NFκB to Induce Myofibroblast Differentiation

**DOI:** 10.3390/biom15101441

**Published:** 2025-10-12

**Authors:** Patrick M. Suess, Chanel C. La, Sreeparna Vappala, Jayachandran N. Kizhakkedathu, James H. Morrissey

**Affiliations:** 1 Department of Biological Chemistry, University of Michigan Medical School, Ann Arbor, MI 48109, USA; 2 Centre for Blood Research, Life Sciences Institute, University of British Columbia, Vancouver, BC V6T 1Z3, Canadajay@pathology.ubc.ca (J.N.K.); 3 Department of Chemistry, University of British Columbia, Vancouver, BC V6T 1Z3, Canada; 4 The School of Biomedical Engineering, University of British Columbia, Vancouver, BC V6T 1Z3, Canada; 5 Department of Pathology and Laboratory Medicine, University of British Columbia, Vancouver, BC V6T 1Z3, Canada

**Keywords:** blood platelets, chemotaxis, fibroblasts, myofibroblasts, platelet activation

## Abstract

Myofibroblasts drive wound healing and fibrotic disease through generation of contractile force to promote wound closure and production of matrix proteins to generate scar tissue. Platelets secrete many pro-wound healing molecules, including cytokines and growth factors. We previously reported that inorganic polyphosphate, secreted by activated platelets, is chemotactic for fibroblasts and induces a myofibroblast phenotype. Using NIH-3T3 cells and primary human fibroblasts, we examined the impact of inhibitors of cell-surface receptors and intracellular signaling molecules on polyphosphate-induced myofibroblast differentiation. We now report that polyphosphate-induced differentiation of fibroblasts to myofibroblasts occurs through a signaling pathway mediated by the receptor for advanced glycation end products (RAGE) and nuclear factor kappa B (NFκB) transcription factor. Inhibition of these signaling components ablated the effects of polyphosphate on fibroblasts. Platelet releasates also induced NFκB signaling and myofibroblast differentiation. Blocking the polyphosphate content of platelet releasates with a biocompatible polyP inhibitor rendered the releasates unable to induce myofibroblast differentiation. These results identify a cell-surface receptor and intracellular transcription factor utilized by platelet polyphosphate to promote wound healing through myofibroblast differentiation and may provide targets for promoting wound healing or altering the disease progression of fibrosis.

## 1. Introduction

Inorganic polyphosphate (polyP), found in all kingdoms of life, consists of linear chains of phosphates linked by high-energy phosphoanhydride bonds [1]. PolyP has been studied most extensively in prokaryotes and unicellular eukaryotes, where it has roles in virulence, biofilm formation, stress endurance, phosphate storage, heavy metal resistance, migration, germination, and proliferation [2,3,4,5,6,7,8,9,10]. Investigating the roles of polyP in mammalian cells has been hindered by the lack of identification of the enzymes responsible for synthesizing polyP, and therefore polyP synthesis cannot currently be blocked by genetic means. Despite this, extracellular polyP has recently emerged as an important regulatory and signaling molecule. Platelets store polyP in their dense granules and secrete this molecule upon platelet activation [11], while other mammalian cell types have also been shown to secrete polyP under a variety of circumstances [12,13]. In mammals, polyP has now been shown to play (or has been implicated in playing) roles in blood coagulation, inflammation, differentiation, migration, biomineralization, and wound healing [11,14,15,16,17,18,19,20,21,22,23].

We previously reported that polyP secreted by activated platelets acts as a signaling molecule to recruit fibroblasts via chemotaxis, and to induce them to transition to myofibroblasts [23]. We proposed that this could play an important role in the normal process of wound healing, in which platelet activation at a wound site not only participates in conferring hemostasis in the short term, but also serves, via polyP secretion, to recruit nearby fibroblasts into the wound site to initiate scar formation locally. Myofibroblasts are contractile cells that promote wound closure and resolution through contractile force and the secretion of matrix proteins, which are essential components of wound healing. On the other hand, myofibroblasts can also drive fibrotic disease through the overaccumulation of pathologic levels of scar tissue in otherwise healthy organs such as lung, liver and bone marrow [24,25]. In the United States, chronic wounds affect over 6 million individuals and place a large financial burden on the healthcare industry [26,27]. It has been estimated that fibrotic diseases are responsible for 45% of all deaths in the industrialized world; however, no cure exists, and treatments remain largely ineffective [28].

Here we report that extracellular polyP induces differentiation in fibroblasts through a signaling pathway mediated by the receptor for advanced end glycation products (RAGE) and nuclear factor kappa B (NFκB). This signaling is required for fibroblasts to accumulate alpha-smooth muscle actin (α-SMA) and several matrix proteins, which are hallmarks of myofibroblast differentiation.

## 2. Materials and Methods

### 2.1. Materials

Antibody to α-SMA (14-9760-82), goat anti-mouse IgG_2a_ secondary antibody (A-21133), Dulbecco’s Modified Eagle Medium (DMEM), ProLong Gold Antifade Mountant, TRIzol, SYBR Green I, bovine calf serum (BCS), bovine serum albumin (BSA), 4-(2-hydroxyethyl)piperazine-1-ethanesulfonic acid (HEPES), Tween-20, and Triton X-100 were from Thermo Fisher Scientific (Waltham, MA, USA). Antibodies to phospho-NFκB p65 (Ser536) (3033) and the alpha-1 chain of type I collagen (COL1A1; 84336) were from Cell Signaling Technology (Danvers, MA, USA). The PAR-1 selective activating peptide, TRAP (S1820), prostaglandin E1, theophylline, and FPS-ZM1 were from Sigma-Aldrich (St. Louis, MO, USA). Cy3 labeled goat anti-rabbit secondary antibody was from Jackson ImmunoResearch Labs (West Grove, PA, USA). JSH-23 was from Abcam (Cambridge, UK). Bengamide B was from Tocris Bioscience (Bristol, UK). 384-well PCR plates were from Bio-Rad (Hercules, CA, USA). Luna Universal One-Step RT-qPCR Kit was from New England Biolabs (Ipswich, MA, USA). The dendrimer-like polyP inhibitor, MPI 8, was synthesized and characterized as described [29].

Heterogeneous preparations of approximately platelet-sized polyP (polyP_75_) and long-chain polyP (polyP_700_) were prepared as previously described [30], except that polyP polymer lengths were estimated by polyacrylamide gel electrophoresis in reference to DNA ladders, as described [31]. In this paper, polyP concentrations are given in terms of the concentration of phosphate monomer.

### 2.2. Cell Culture

Fibroblast growth medium (termed here, supplemented DMEM) consisted of DMEM containing 4 mM L-glutamine, 10 mM streptomycin, 100 I.U./mL penicillin, 25 mM HEPES, plus the indicated concentration of BCS. The established mouse fibroblast line, NIH-3T3 (American Type Culture Collection, ATCC CRL-1658), and primary fetal human lung fibroblasts, GM05387 (Coriell Institute, NIGMS Human Genetic Cell Repository), were routinely cultured at 37 °C in a 5% CO_2_ environment in supplemented DMEM (10% BCS). Cells were cultured for no more than 2 weeks at a time and maintained at less than 90% confluency.

### 2.3. Immunofluorescence

For immunofluorescence studies, cells were allowed to attach for 24 h on glass coverslips in supplemented DMEM (2.5% BCS), seeded at 1.6 × 10^4^ cells/cm^2^ if cells were to be stimulated 1 h, or at 0.5 × 10^4^ cells/cm^2^ if cells were to be stimulated 48 h. The next day, the medium was replaced with supplemented DMEM (1.5% BCS) plus the desired stimulant for 1 or 48 h, after which the cells were fixed, permeabilized and stained at room temperature as follows. First, cells on cover slips were washed once with TBS (50 mmol/L Tris-HCl pH 7.5, 150 mmol/L NaCl), then fixed for 10 min with 4% paraformaldehyde in TBS followed by three TBS washes. Fixed cells were then permeabilized with 0.2% Triton X-100 in TBS for 5 min, followed by three TBS washes and 1 h blocking with 0.05% Tween-20, 5% BSA in TBS. Cells were then incubated 1 h with primary antibody in 0.05% Tween-20, 1% BSA in TBS, washed three times with TBS, and then incubated 1 h in the dark with secondary antibody and SYBR Green 1 (to stain DNA; 1:500,000 dilution) in 0.05% Tween-20, 1% BSA in TBS. Cells were washed three times with TBS, briefly rinsed with water, mounted onto glass microscope slides with Antifade Mountant, and allowed to sit overnight at room temperature, after which they were stored dark at 4 °C until imaging. Primary antibodies were diluted 1:1000 for phospho-NFκB, 1:1000 for α-SMA, and 1:500 for COL1A1. Secondary antibodies were diluted 1:500. Images were obtained on a Stellaris 5 confocal microscope (Leica Microsystems, Wetzlar, Germany). A minimum of 50 individual cells from a minimum of 5 representative images were analyzed for each experimental condition. Integrated density (defined as product of cell area and mean fluorescence intensity) was determined using ImageJ version 1.53 and the average integrated density for each experiment was determined [32].

### 2.4. Reverse Transcription Quantitative Polymerase Chain Reaction (RT-qPCR)

NIH-3T3 cells were stimulated with polyP in the presence or absence of the indicated inhibitors for 1 h in supplemented DMEM (1.5% BCS) and RNA was immediately extracted using TRIzol according to the manufacturer’s instructions. One ng of RNA was used as template for RT-qPCR with 150 nM of each primer, using a CFX384 Touch Real-Time PCR Detection System (Bio-Rad). The housekeeping gene, ribosomal protein L13a (RPL13A), was used as the reference target. Primers were from Integrated DNA Technologies (Newark, NJ, USA), as follows (5′ to 3′): transforming growth factor beta 1 (TGF-β1) forward: ACAGGGCTTTCGATTCAGCG, reverse: GGAAGGGCCGGTTCATGTC; alpha-1 chain of type II collagen (COL2A1) forward: TATCTGTGAAGACCCAGACTGCC, reverse: CCTTTGGCCCTAATTTTCCACTG; alpha-1 chain of type III collagen (COL3A1) forward: CACGTAAGCACTGGTGGACA, reverse: CAGGAGGGCCATAGCTGAAC; fibronectin (FN1) forward: GCCACCATTACTGGTCTGGAG, reverse: GGGGTGTGGAAGGGTAACCA; and RPL13A forward: CTGAAGCCTACCAGAAAGTTTGC, reverse: CACTGCCTGGTACTTCCACC. Fold changes in transcription levels were calculated as 2^−(−ΔΔCt)^, as described [33].

### 2.5. Platelet Releasates

Recently expired human platelet units were kindly provided without patient identifying information by the University of Michigan Blood Bank. Platelet releasates were prepared as described [23].

## 3. Results

### 3.1. Role of NFκB in the Response of Fibroblasts to PolyP

**Figure 1:**
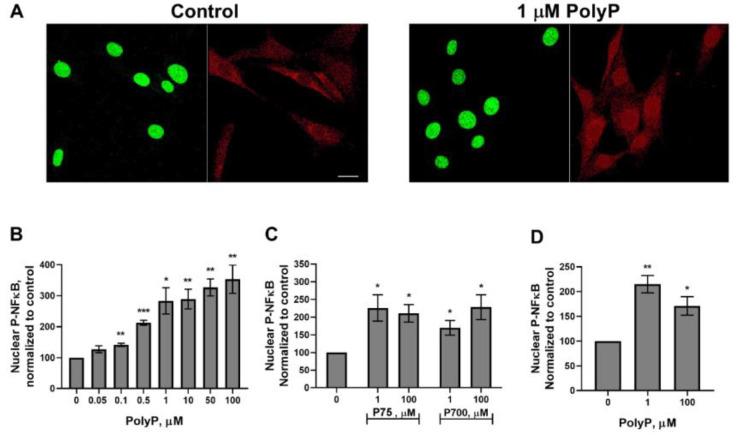
PolyP induces nuclear translocation of phospho-NFκB in fibroblasts. (**A**) Representative confocal microscopy images of NIH-3T3 cells incubated 1 h without (Control) or with 1 μM polyP_75_, then stained for DNA (green) and phospho-NFκB (red); bar = 20 µm. (**B**) Integrated intensity of phospho-NFκB (P-NFκB) staining in NIH-3T3 cell nuclei as a function of polyP_75_ concentration (after 1 h incubation), normalized to cells not exposed to polyP (with the normalized signal set at 100). (**C**) Normalized intensity of nuclear phospho-NFκB staining in NIH-3T3 cells exposed for 1 h to the indicated concentrations of polyP_75_ or polyP_700_. (**D**) Normalized intensity of nuclear phospho-NFκB staining in GM05387 cells exposed for 1 h to the indicated concentrations of polyP_75_. Nuclear antibody staining for phospho-NFκB was quantified by confocal microscopy, and all values are mean ± standard error of the mean (SEM; *n* = 5). Asterisks highlight statistical significance relative to the no-polyP controls: * *p* < 0.05, ** *p* < 0.01, *** *p* < 0.001 (paired *t*-tests).

Platelet-derived polyP has been shown to activate endothelial cells via NFκB signaling [16,34]. We therefore examined the role of NFκB in the responses of fibroblasts to polyP by incubating NIH-3T3 cells with platelet-sized polyP, after which the levels of phospho-NFκB in nuclei were assessed. We found that physiologically relevant (low micromolar) concentrations of polyP induced nuclear translocation of phospho-NFκB in NIH-3T3 cells (Figure 1A,B). PolyP can have differential effects based on size (polymer length) in some mammalian systems [35,36]. We found that both platelet-sized and long-chain polyP induced nuclear localization of phospho-NFκB (Figure 1C). NIH-3T3 cells are an immortalized mouse cell line with fibroblast properties [37], so to extended this study to primary human fibroblasts we repeated selected experiments with primary human fetal lung fibroblasts (GM05387 cells [38]). When incubated with polyP we found, as with NIH-3T3 cells, that micromolar concentrations of polyP induced phospho-NFκB accumulation in nuclei of GM05387 cells (Figure 1D). These data indicate that in addition to endothelial cells, polyP induces NFκB signaling in fibroblasts.

**Figure 2:**
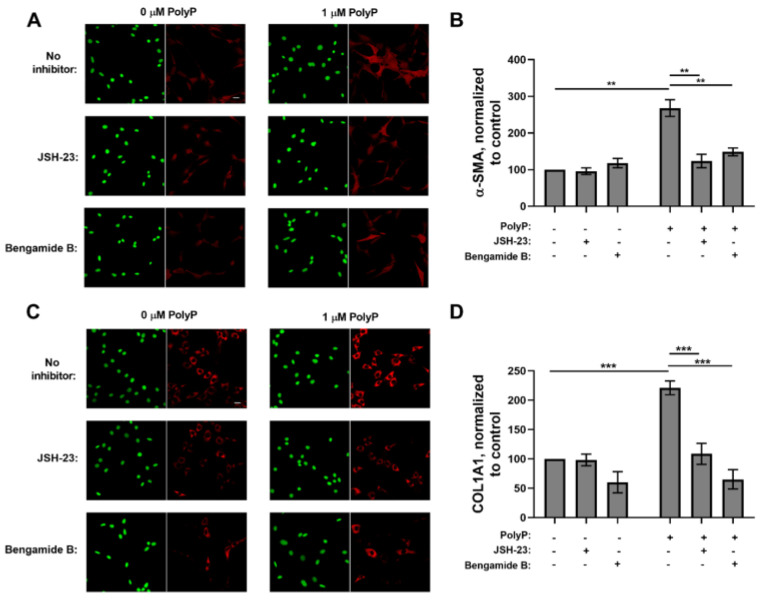
NFκB inhibitors block myofibroblast differentiation induced by polyP in NIH 3T3 cells. NIH-3T3 cells were incubated for 48 h in 1.5% BCS without or with 1 µM polyP_75_, in the presence or absence of the NFκB inhibitors, JSH-23 (10 µM) or Bengamide B (100 nM), after which antibody staining was performed. (**A**) Representative images after staining for DNA (green) and α-SMA (red); bar = 20 µm. (**B**) Normalized staining intensity for α-SMA in cells incubated without or with polyP_75_, in the presence or absence of JSH-23 or bengamide B, as indicated. (**C**) Representative images after staining for DNA (green) and COL1A1 (red); bar = 20 µm. (**D**) Normalized staining intensity for COL1A1 in cells incubated without or with polyP_75_, in the presence or absence of JSH-23 or bengamide B, as indicated. All values are mean ± SEM (*n* = 5). Asterisks highlight statistical significance of the indicated comparisons: ** *p* < 0.01, *** *p* < 0.001 (paired *t*-tests).

We previously reported that polyP induces myofibroblast differentiation in murine and human fibroblasts [23]. Since NFκB is known to promote myofibroblast differentiation and survival [39,40], we investigated whether polyP-induced differentiation of fibroblasts into myofibroblasts required the participation of NFκB. Accordingly, we cultured NIH-3T3 cells with polyP in the presence or absence of the NFκB inhibitors, JSH-23 [41] or Bengamide B [42], then assessed two markers of myofibroblast differentiation: α-SMA and COL1A1. Both of the NFκB inhibitors abrogated the polyP-induced increase in α-SMA (Figure 2A,B) and COL1A1 (Figure 2C,D) in these cells. This indicates that signal transduction via NFκB is essential for polyP to induce the differentiation of fibroblasts into the myofibroblast phenotype.

### 3.2. RAGE Mediates the Fibroblast Response to PolyP

**Figure 3:**
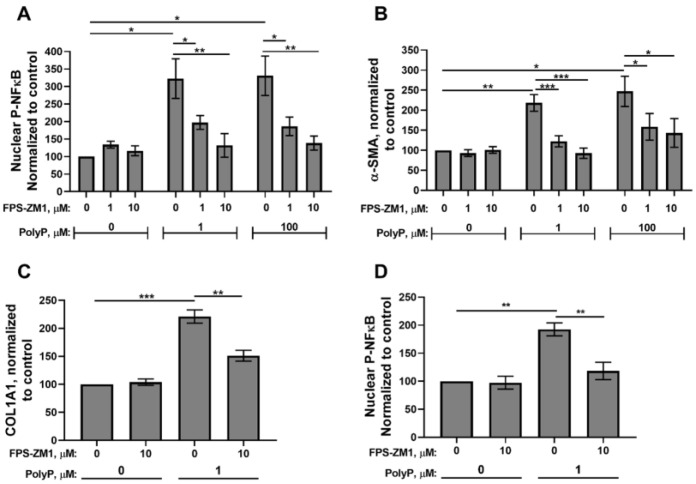
RAGE inhibitors diminish polyP-induced differentiation of fibroblasts into a myofibroblast phenotype, and reduce phospho-NFκB accumulation in their nuclei. (**A**) NIH-3T3 cells were incubated for 1 h in 1.5% BCS with the indicated concentration of polyP_75_ and the RAGE antagonist, FPS-ZM1, then stained for phospho-NFκB, after which nuclear antibody staining for phospho-NFκB was quantified. (**B**,**C**) NIH-3T3 cells were incubated for 48 h in 1.5% BCS with the indicated concentrations of polyP_75_ and FPS-ZM1, then stained for the indicated antigens, quantified using confocal microscopy. (**D**) GM05387 cells were incubated for 1 h in 1.5% BCS with the indicated concentrations of polyP_75_ and FPS-ZM1, after which nuclear antibody staining for phospho-NFκB was quantified. All values are mean ± SEM (*n* = 5). Asterisks highlight statistical significance of the indicated comparisons: * *p* < 0.05, ** *p* < 0.01, *** *p* < 0.001 (paired *t*-tests).

Although the receptors utilized by polyP for cellular signaling in mammalian systems are relatively little explored, recent studies have demonstrated that the purinergic receptor, P2Y_1_, and the receptor for advanced glycation end products (RAGE) can act as polyP cell surface receptors on endothelial, neuronal, and fibroblastic cells [23,43,44]. We previously reported that polyP-induced differentiation of fibroblasts into myofibroblasts was independent of P2Y_1_ signaling [23]. We therefore assessed whether polyP-induced myofibroblast differentiation was dependent on RAGE. We now report that the high-affinity RAGE antagonist, FPS-ZM1 [45], abrogated the nuclear translocation of phospho-NFκB in polyP-treated NIH-3T3 cells (Figure 3A). RAGE antagonism also inhibited the accumulation of two markers of myofibroblast differentiation, α-SMA and COL1A1, in NIH-3T3 cells (Figure 3B,C). Furthermore, RAGE inhibition also abrogated NFκB activation in primary human fibroblast cells (GM05387 cells; Figure 3D). These studies indicate that extracellular polyP signaling in fibroblasts is mediated by RAGE, leading to NFκB activation and myofibroblast differentiation.

**Figure 4:**
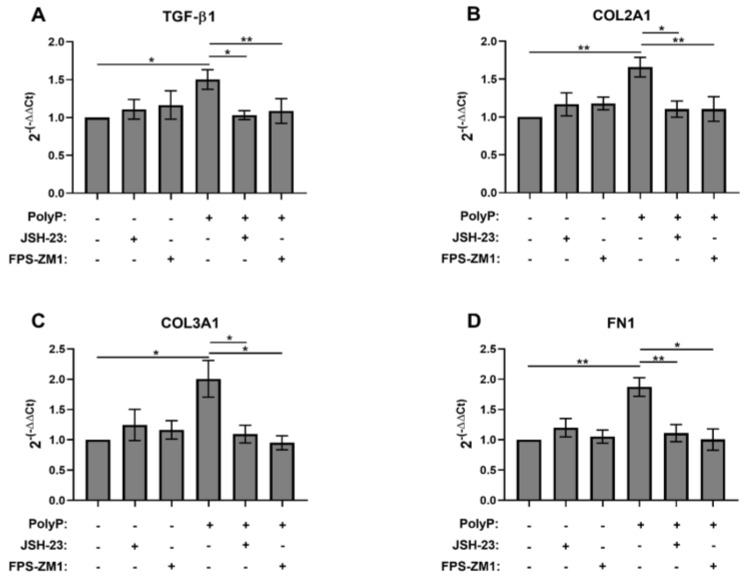
Effect of RAGE and NFκB inhibitors on polyP-induced transcription of genes for TGF-β1and the matrix proteins, COL2A1, COL3A1, and FN1. NIH-3T3 cells were incubated in 1.5% BCS with 1 µM polyP_75_ in the presence or absence of 10 µM JSH-23 or 1 µM FPS-ZM1 for 1 h and RNA was extracted and used as a template for RT-qPCR for the indicated genes (**A**–**D**). All values are mean ± SEM (*n* = 5). Asterisks highlight statistical significance of the indicated comparisons: * *p* < 0.05, ** *p* < 0.01 (paired *t*-tests).

We next assessed transcript levels of additional myofibroblast markers induced by polyP using RT-qPCR with RNA from NIH-3T3 cells stimulated with platelet-sized polyP. We also examined the dependency of these transcripts on signaling via RAGE and NFκB. We found that 1 μM polyP increased the transcript levels of TGF-β1 (known to be a major driver of myofibroblast differentiation [46,47]) as well as the transcript levels of the matrix proteins, COL2A1, COL3A1, and FN1 (Figure 4A–D). Inhibitors of RAGE and NFκB abrogated the polyP-mediated induction of transcripts for all of these proteins (Figure 4A–D).

**Figure 5:**
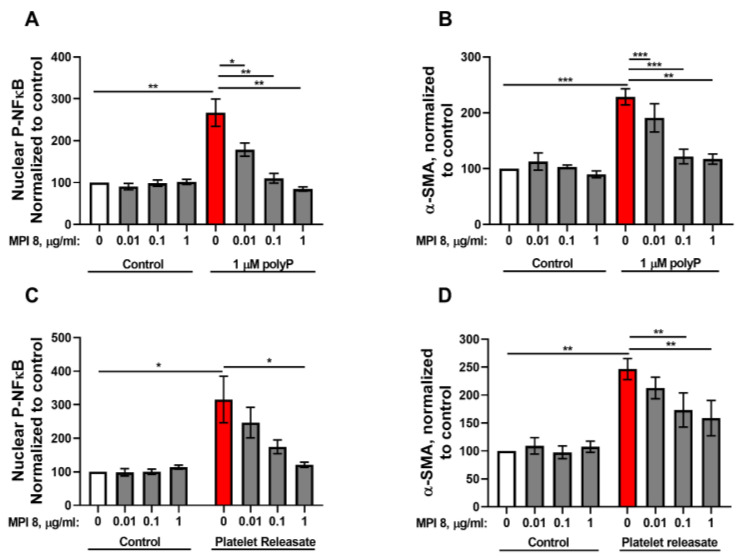
The polyP inhibitor, MPI 8, abrogates fibroblast differentiation and phospho-NFκB nuclear translocation induced by synthetic polyP or by platelet releasates. (**A**,**B**) NIH-3T3 cells were incubated in 1.5% serum with 1 µM polyP_75_ in the presence or absence of the indicated concentration of MPI 8. (**A**) After one hour, nuclear antibody staining for phospho-NFκB was quantified using confocal microscopy. (**B**) After 48 h antibody staining for α-SMA was quantified using confocal microscopy. (**C**,**D**) NIH-3T3 cells were incubated in 1.5% serum with releasates from activated human platelets (diluted to 15 μg/mL protein) in the presence or absence of the indicated concentration of MPI 8. (**C**) After one hour, nuclear antibody staining for phospho-NFκB was quantified using confocal microscopy. (**D**) After 48 h antibody staining for α-SMA was quantified using confocal microscopy. All values are mean ± SEM (*n* = 5). Asterisks highlight statistical significance of the indicated comparisons: * *p* < 0.05, ** *p* < 0.01, *** *p* <0.001 (paired *t*-tests).

We previously reported the synthesis and characterization of a family of highly biocompatible, dendrimer-like cationic compounds, including agents that safely and potently reverse the anticoagulant activity of heparin [48,49,50], or block the prothrombotic activity of polyP [50,51,52], both in vitro and in vivo. Our newest-generation polyP inhibitor, MPI 8, is an especially potent polyP inhibitor while showing even better biocompatibility than our earlier-generation compounds [29]. In this study, we tested the ability of MPI 8 to abrogate myofibroblast differentiation of NIH-3T3 cells induced by platelet-sized polyP and found that 1 μg/mL MPI 8 drastically reduced NFκB activation and α-SMA accumulation in these cells (Figure 5A,B). We previously showed that releasates harvested from activated human platelets will induce differentiation of fibroblasts into myofibroblasts (visualized by α-SMA accumulation), and by using a polyP-degrading enzyme we further demonstrated that the polyP content of such releasates was the major activating component [23]. We now report that platelet releasates promoted NFκB activation in NIH-3T3 cells, and that this activity was completely abrogated by adding 1 μg/mL MPI 8 to the releasates (Figure 5C). Similarly, we found that adding 1 μg/mL MPI 8 to platelet releasates severely blunted their ability to induce α-SMA accumulation in NIH-3T3 cells (Figure 5D). These results suggest that polyP released from the dense granules of activated platelets induces NFκB signaling to promote myofibroblast differentiation, and that the use of MPI 8 is a unique way of blocking the action of polyP, even in complex mixtures like platelet releasates.

## 4. Discussion

Platelet-secreted polyP is an emerging cell signaling molecule with activity reported for endothelial cells, neutrophils, monocytes, macrophages, osteoblasts, neurons, astrocytes [15,17], and keratinocytes [19,20,21,43,44]. Despite these numerous findings, the signaling mechanisms utilized by polyP have not been extensively studied. Platelet polyP has previously been shown to activate NFκB in endothelial cells in a pathway mediated by the RAGE and P2Y_1_ receptors [43], while NFκB signaling has also been linked to myofibroblast differentiation [53]. Activation of RAGE by other ligands has also been shown to activate fibroblast proliferation and induce fibrosis [54,55]. Here, we show that polyP promotes myofibroblast differentiation of fibroblasts in a signaling pathway mediated by RAGE and NFκB. Platelet-sized polyP induced NIH-3T3 cell nuclear translocation of phospho-NFκB in a concentration range that matches polyP-induced myofibroblast differentiation [23]. Like polyP-induced myofibroblast differentiation, both short and long-chain polyP induce NFκB activation [23]. PolyP caused fibroblasts to increase the levels of protein and/or mRNA for α-SMA, TGF-β1, fibronectin and types I, II and III collagen, while inhibition of RAGE or NFκB signaling abrogated these effects of polyP on fibroblasts.

Chronic wounds such as diabetic ulcers represent a large burden to millions of patients and present financial burdens to the healthcare industry in the United States [26]. Platelets produce components that promote wound healing [56,57], and exogenously applied polyP has been shown to promote the wound healing process in vivo [18,21]. In our previous study we showed that it was the polyP component of platelet releasates that was essential for promoting the differentiation of fibroblasts into a myofibroblast phenotype [23], while in the present study we extended these observations to show that platelet releasates promote NFκB activation, revealing more of the molecular mechanism by which polyP and platelets modulate this process. We also show that the highly biocompatible, dendrimeric polyP inhibitor MPI 8 can effectively block the differentiation-inducing action of polyP in platelet releasates.

The mechanism by which polyP elicits signaling via RAGE in fibroblasts is not yet clear. A previous study by Dinarvand et al. [43] with cultured endothelial cells found that direct signaling of polyP via RAGE required very high polyP concentrations (50 μM), but when endothelial cells were incubated with polyP together with either HMGB1 or histone H4, the required polyP concentration dropped to just 2.5 μM (similar to the polyP concentrations we found to elicit RAGE-dependent signaling in fibroblasts). They also showed that polyP binds with high affinity to both HMGB1 and histone H4. Their findings indicated that it was likely that complexes of polyP with HMGB1 or histone H4 (which are known RAGE ligands) were much more effective at inducing signals than was polyP alone. On the other hand, Dinarvand et al. found that this signaling also required P2Y_1_ and provided evidence that polyP/HMBG1 complexes signaled in endothelial cells in a mechanism in which the polyP bridged between both RAGE and P2Y_1_. Not surprisingly, this required very long-chain polyP [43]. We previously showed that the ability of polyP to elicit differentiation of fibroblasts into myofibroblasts was independent of P2Y_1_, and furthermore that it was maximal at polyP polymer lengths equivalent to platelet-sized polyP [23]. Although the mechanism of polyP signaling through RAGE on fibroblasts appears to be different from that of endothelial cells, it nevertheless seems possible that the true ligand that is binding to RAGE in our studies could be a complex of polyP and another protein. Since our experiments were conducted in the presence of calf serum, it is possible that polyP could be activating RAGE via association with such a serum protein. It is also possible that polyP is interacting with a protein secreted from fibroblasts (such as TFG-β), amplifying an autocrine signaling mechanism. Examining these ideas is beyond the scope of the present study but should be addressed in future research.

Further investigation of polyP as a potential therapeutic for chronic wounds is warranted. While myofibroblasts are essential for the wound healing process by promoting wound closure, wound resolution, and scar tissue production, this cell type is also the primary effector cell in fibrotic diseases [58]. Platelet polyP also promotes differentiation in vitro of another pro-fibrotic cell type, the fibrocyte [20]. The possible role of platelet-derived polyP in various pathologies has yet to be investigated, which can largely be attributed to a lack of genetic means by which to inhibit polyP synthesis in mammalian organisms. MPI 8 may therefore be a useful tool with which to inhibit the effects of extracellular polyP in vivo, including examining the potential role of platelet-derived polyP in inflammation, thrombosis and fibrotic diseases [50,52]. Myelofibrosis in bone marrow is thought to be driven, at least in part, by megakaryocytes, the cells responsible for platelet production in the bone marrow [59,60]. Idiopathic pulmonary fibrosis patients have been shown to have increased platelet reactivity and activation attributed to an unknown plasma factor [61]. MPI 8 should allow for investigation of the role of polyP in vivo in such disease states.

## 5. Conclusions

This study shows that polyP promotes differentiation of fibroblasts in vitro into a myofibroblast phenotype via a signaling pathway mediated by RAGE and NFκB. It also shows that polyP induces fibroblasts to increase production of α-SMA, TGF-β1, fibronectin and types I, II and III collagen, also in a manner that requires RAGE and NFκB signaling. It also shows that a highly biocompatible polyP inhibitor (MPI 8) is an effective tool for blocking the differentiation-inducing action of polyP that is present in complex platelet releasates.

## Data Availability

The original contributions presented in this study are included in the article. Further inquiries can be directed to the corresponding author.

## Abbreviations

α-SMA	alpha-smooth muscle actin
BCS	bovine calf serum
BSA	bovine serum albumin
COL1A1	alpha-1chain of type I collagen
COL2A1	alpha-1 chain of type II collagen
COL3A1	alpha-1 chain of type III collagen
DMEM	Dulbecco’s modified Eagle medium
FN1	fibronectin
HEPES	4-(2-hydroxyethyl)piperazine-1-ethanesulfonic acid
NFκB	nuclear factor kappa B
polyP	polyphosphate
polyP75	platelet-sized polyphosphate
polyP700	long-chain polyphosphate
RAGE	receptor for advanced glycation end products (RAGE)
RPL13A	ribosomal protein L13a
RT-qPCR	reverse transcription quantitative polymerase chain reaction
TBS	50 mmol/L Tris-HCl pH 7.5, 150 mmol/L NaCl
TGF-β1	transforming growth factor beta 1
